# RHOA protein expression correlates with clinical features in gastric cancer: a systematic review and meta-analysis

**DOI:** 10.1186/s12885-022-09904-7

**Published:** 2022-07-19

**Authors:** Seungyoon Nam, Yeeun Lee, Jung Ho Kim

**Affiliations:** 1 Department of Health Sciences and Technology, Gachon Advanced Institute for Health Sciences and Technology (GAIHST), Gachon University, Incheon, 21999 Korea; 2 Department of Genome Medicine and Science, AI Convergence Center for Medical Science, Gachon Institute of Genome Medicine and Science, Gachon University Gil Medical Center, Gachon University College of Medicine, Namdong-daero 774 beon-gil 21, Namdong-gu, Incheon, 21565 Korea; 3 Department of Life Sciences, Gachon University, Seongnam, 13120 Gyeonggi-do Korea; 4 Department of Internal Medicine, Gachon University Gil Medical Center, Gachon University College of Medicine, Incheon, 21565 Korea

**Keywords:** RHOA, Gastric cancer, Meta-analysis, Immunohistochemistry

## Abstract

**Background:**

Gastric cancer (GC) is one of the most fatal cancers worldwide and is generally only detected after it has progressed to an advanced stage. Since there is a lack of comprehensive data on RHOA protein expression of patients with GC, this study utilized a systematic review and meta-analysis to address the limitation. The objective of this meta-analysis was to link GC clinical features with RHOA protein high- vs. low-expressing patients with GC.

**Methods:**

The PubMed and Web of Science were used for a systematic literature review of GC related to RHOA. The included studies were obtained from two literature databases from past to Aug 31, 2021, by searching keywords. This meta-analysis followed the Preferred Reporting Items for Systematic Reviews and Meta-Analysis (PRISMA) guidelines. The odds ratios (ORs) and 95% confidential intervals (CIs) for clinical features were estimated according to the high and low protein expression levels of RhoA. The mean effect sizes of ORs were obtained using the random-effects and fixed-effects models of meta-analysis. Heterogeneity of the studies was assesed by using statistics: τ^2^, I^2^; and Q values. The symmetry of funnel plots were inspected for publication bias.

**Results:**

Finally, 10 studies including 1,389 patients with GC (735 RHOA-positive and 654 RHOA-negative) were eligible for our meta-analysis to estimate associations between the protein expression and clinical features (e.g., Union for International Cancer Control [UICC] stage progression, differentiation, Lauren histological classification, and vascular invasion). In our meta-analysis, RHOA positive expression was determined to have a statistically significant association with UICC stage progression (*P* = 0.02) and poorly differentiated status (*P* = 0.02). The association between RHOA positivity and Lauren subtypes was not statistically significant (*P* = 0.07).

**Conclusions:**

This meta-analysis suggested that RhoA protein expression in patients with GC was associated with clinical features: UICC stage progression and poorly differentiated status. Our findings are inconclusive but indicate that high RHOA protein expressing patients with GC could predict advanced UICC stages. A large prospective cohort study is required for validation in future.

## Background

There were 1,089,103 new cases of gastric cancer (GC) worldwide in 2020, with 768,793 deaths [1]. East Asia, including Korea, Japan, and China, has a higher prevalence of GC than other regions. However, GC therapeutics and biomarkers are yet to be confirmed [2–4].

Ras homologous A (RHOA), a Rho family small GTPase, is involved in diverse oncogenic processes, including proliferation, migration, cell polarity, and invasion [5], as well as microtubule destabilization and epithelial-to-mesenchymal transition (EMT) [5, 6]. RHOA is a biomarker candidate, and also a therapeutic target for GC progression [7, 8].

Previous studies for RHOA in GC have dealt with biological functions and molecular subtypes. A molecular subtype in GC was associated with RHOA genetic events (e.g., DNA mutations, copy number alterations [CNAs]) in the Cancer Genome Atlas project [9, 10]. RHOA mutations were frequently observed in the GC molecular subtype “genomically stable” (GS), which exhibited low CNAs and overlapped with Lauren subtype diffuse GC [9, 11]. In fact, 75% of patients with diffuse subtype GC were assigned to the molecular subtype GS [9]. In siRNA knockdown of *RHOA* in diverse GC cell lines, cell growth was inhibited, and apoptosis increased [7]. In vivo xenograft model of sh*RHOA*, tumor size was decreased [7]. Despite functional importance of RHOA in GC, clinical associations of RHOA protein expression have not been elucidated. The difference between our study and previous studies is that our meta-analysis focused on estimating clinical associations for patients with GC expressing high and low RHOA protein levels.

RHOA has been inspected mainly by individual studies in terms of biological functions, and genetic characterization in GC [5]. RHOA functions including migration and EMT are expected to be associated with the following clinical features: GC progression, invasion, and GC cell histology (i.e., differentiation) [5]. However, there is a lack of demonstrating associations between RHOA protein expression and the GC clinical features related to the RHOA functions. Thus, a quantitative synthesis is necessary to estimate the associations between RHOA protein expression and the clinical features. The systematic reviews and meta-analyses for RHOA inhibitors have been studied in spinal cord injury and ischaemic stroke [12–14], but those for RHOA protein expression not studied in GC.

The objective of this meta-analysis was to estimate associations between GC clinical features and RHOA protein high- vs. low-expressing GC patients. The clinical utility of RHOA protein expression in patients with GC have not been reported. Thus, this systematic review and meta-analysis summarizes previous publications. This approach inspects whether RHOA protein expression predicts clinical features including GC progression, invasion, and GC cell histology. This comprehensive analysis helps to demonstrate the possible clinical associations of RHOA protein expression and the mean effect sizes of the clinical features in patients with GC by synthesizing evidence from the publications.

## Materials and methods

### Literature inspection

**Fig. 1:**
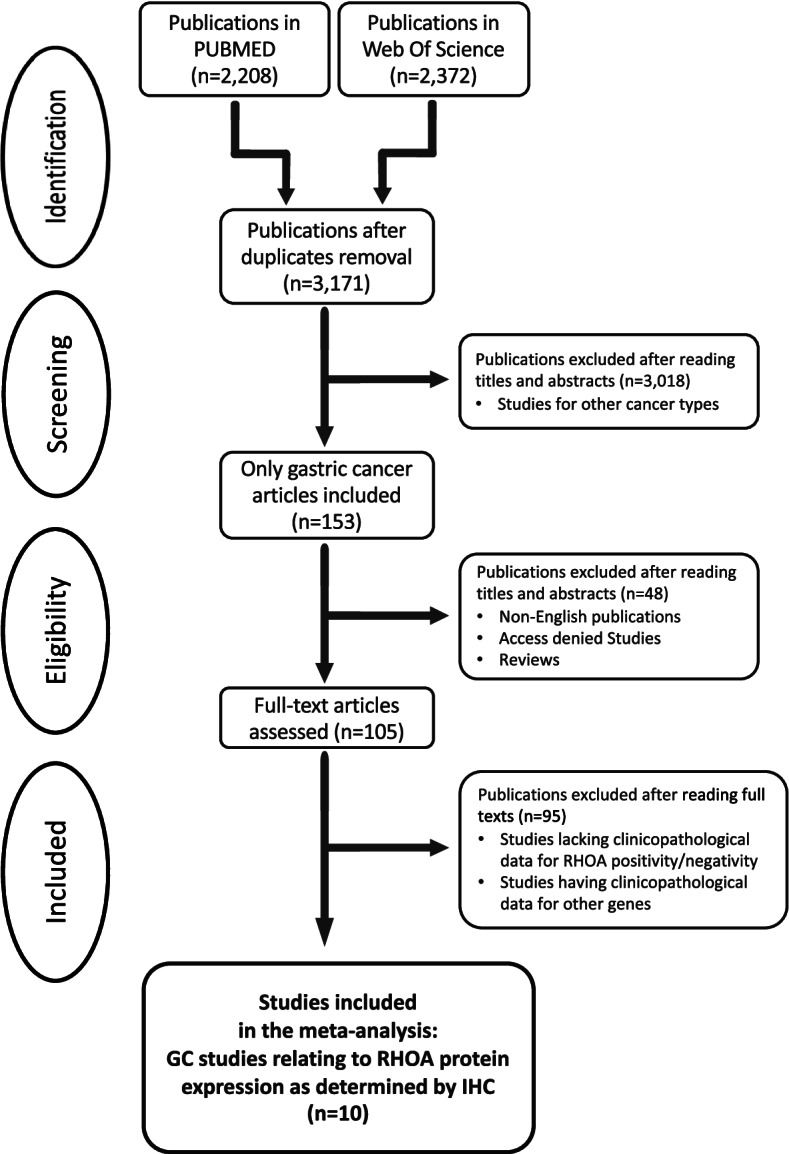
Flow of systematic publication selection processes

This study was performed according to the Preferred Reporting Items for Systematic Reviews and Meta-Analysis (PRISMA). PubMed and Web of Science were searched to obtain literatures on RHOA expression in GC, in order to identify appropriate publications for a meta-analysis, through Aug 31, 2021, with the following terms: “RHOA,” “cancer,” and “expression.” Subsequently, the titles and abstracts of the publications that contained term “gastric” were retrieved (Fig. 1).

### Selection criteria

Included studies were selected by the following exclusion and inclusion criteria.

#### Inclusion criteria:

- 1. GC studies relating to RHOA protein expression as determined by immunohistochemistry (IHC)
- 2. Studies relating to pathological diagnosis of GC
- 3. Studies of case–control design in GC
- 4. Studies with sufficient data to derive odds ratios (ORs) and 95% confidence intervals (CIs).
- 5. Articles published between the past and Aug 31, 2021.

#### Exclusion criteria

- 1. Reviews
- 2. Non-English publications
- 3. Access denied studies
- 4. Studies on cancers other than GC
- 5. Studies lacking clinicopathological data for RHOA positive and negative protein expression
- 6. clinicopathological studies for other genes.

### Type of studies

The included studies were retrospective case–control studies that compared high- and low-RHOA protein expressing patients with GC. These studies had clinical information (i.e., sex, age, Union for International Cancer Control [UICC] TMN stage progression, UICC T classification, UICC M classification, UICC N classification, Lauren classification, differentiation, tumor sites, Bormann types, lymphatic invasion, neural invasion, and vascular invasion).

### Data extraction

To obtain relevant information for our meta-analysis, the two author reviewers (SN and YL) independently assessed the literature that satisfied the two previous criteria. Authors, publication year, study objects, RHOA positivity, technique, and clinical information were included (Table 1).

**Table 1:** The ten included publications for RHOA protein expression for meta-analysis. In the studies, the RHOA protein expression was measured by immunohistochemistry (IHC). The RHOA antibodies used for IHC were described. The quantification method (i.e., IHC score) of the RHOA protein expression in each study was summarized. Study names for meta-analysis are indicated in parentheses in the first column

Publications (study names)	IHC score description	Antibodies
Zhou et al. [15] (Zhou, 2011)	Both the proportion of positive cells and staining intensity were used to measure RHOA	Anti-RhoA (Santa Cruz Biotechnology, TX, USA)
Yoon et al. [16] (Yoon, 2016)	The staining intensity was multiplied by the staining extent to obtain a RHOA score	Anti-RhoA (ab54835; Abcam, Cambridge, UK); anti-phosphorylated-RhoA (ab125275; Abcam)
Liu et al. [17] (Liu, 2019)	The RHOA score was obtained by multiplying staining extent score by intensity score	Anti-RhoA (SAB1400018; Sigma-Aldrich)
Korourian et al. [18] (Korourian, 2017a)	The RHOA score was obtained by multiplying the intensity of staining by the proportion of positive tumor cells	Anti-RhoA (ab54835, Abcam)
Lin et al. [19] (Lin, 2007)	The RHOA score was classified by the extent of cell stained	Anti-RhoA (Santa Cruz Biotechnology)
Chang et al. [20] (Chang, 2016)	The RHOA score was calculated by multiplying the staining intensity by the staining extent	Anti-phosphorylated-RhoA (ab125275; Abcam)
Korourian et al. [21] (Korourian, 2017b)	Histochemical score (H-score) was obtained by multiplying the staining intensity by the proportion of positive tumor cells	Anti-RhoA (ab54835, Abcam)
Liu et al. [22] (Liu, 2004)	The ratio of positive cells and staining intensity were evaluated	Anti-RhoA (Santa Cruz Biotechnology)
Xu et al. [23] (Xu, 2019)	RHOA was measured by the proportion of stained tumor cells	Anti-RhoA (clone 26C4; Santa Cruz Biotechnology)
Huang et al. [24] (Huang, 2015)	Expression was obtained by the proportion of stained tumor cells	Anti-RhoA (clone 26C4; Santa Cruz Biotechnology)

The author (SN) reviewed the whole search, and confirmed the data.

### Quality assessment

Recommendations and quality of evidence of the included studies were evaluated according to the guidelines of Robinson et al. [25] (Table 2).

**Table 2:** Evaluation of quality of evidence for our included studies according to the guidelines of Robinson et al. [25]. (“2A: weak recommendation; limited quality; patient-oriented evidence. B: Systematic review/meta-analysis of lower quality cohort studies with inconsistent results that may vary depending on circumstances or patients or societal values; retrospective cohort studies; case–control study. C: consensus guidelines; usual practice; expert opinion; case series; other alternatives may be equally reasonable [25].”)

Authors (study names)	Publication Year	Grade of Recommendation	Quality of Evidence
Zhou et al. [15] (Zhou, 2011)	2011	2A	B
Yoon et al. [16] (Yoon, 2016)	2016	2A	B
Liu et al. [17] (Liu, 2019)	2019	2A	B
Korourian et al. [18] (Korourian, 2017a)	2017	2A	B
Lin et al. [19] (Lin, 2007)	2007	2A	C
Chang et al. [20] (Chang, 2016)	2016	2A	B
Korourian et al. [21] (Korourian, 2017b)	2017	2A	B
Liu et al. [22] (Liu, 2004)	2004	2A	B
Xu et al. [23] (Xu, 2019)	2019	2A	C
Huang et al. [24] (Huang, 2015)	2015	2A	C

### Assessment of heterogeneity and statistics

The R library "meta" [26] was utilized for this meta-analysis, generating forest plots using ORs and their 95% CIs from the included publications.

The pooled effect sizes of the ORs were estimated using either random-effects models or fixed-effects models (equivalently, common-effects model). The pooled effect size of the OR is a critical tool in assessing the clinical relevance of RHOA protein high- vs. low-expressing patients with GC and refers to the collective effect size estimates of the studies.

The heterogeneity was assessed using statistics (between-study variance τ^2^ and Higgins’ I^2^ including Cochran’s Q-tests) and was obtained by the R library "meta" [26]. We used fixed-effects (equivalently, common-effects) models to produce pooled ORs when I^2^ ≤ 50% or P ≥ 0.05 showed the absence of heterogeneity [27]. Otherwise, pooled ORs were calculated using random-effects models [27, 28]. The forest plots were generated to demonstrate the clinical outcomes of RHOA protein high- vs. low-expressing patients with GC.

In terms of biological functions, RHOA overexpression is important in cell migration and cell proliferation of cancer [29, 30]. But, how RHOA protein expression affects clinical features relating to cancer cell proliferation and migration was not systematically inspected. Cancer cell proliferation and migration promote advanced cancer stages, and cytology. In the line, the clinical features including UICC TMN stage progression and cancer cell differentiation status were inspected. Additional clinical features (i.e., sex, age, UICC T classification, UICC M classification, UICC N classification, Lauren classification, tumor sites, Bormann types, lymphatic invasion, neural invasion, and vascular invasion) were also inspected.

### Sensitivity analysis and publication bias

To measure the effects of individual studies on the overall conclusions for the statistically significant clinical features, sensitivity analysis was performed by individually deleting each study.

Next, publication bias was assessed by using funnel plots (standard error of OR vs. OR). In visual inspection of funnel plots, lack of skewness and asymmetry generally indicates an absence of publication bias.

## Results

### Included studies and their information

The search of PubMed and Web of Science generated 2,208 and 2,372 studies, respectively. After removing duplicates and carefully reviewing the titles and abstracts of the studies, 105 studies were found. Subsequently, the 105 studies were thoroughly reviewed, and 95 were eliminated due to a lack of data and an unclear number of patients. Resultingly, ten publications were selected for meta-analysis (Table 1). Figure 1 depicts the PRISMA flow. IHC was performed to determine RHOA expression (Table 1). The 10 publications had a total of 1,389 patients, with 735 RHOA-positive and 654 RHOA-negative GC patients. The included studies in our meta-analysis should have RHOA protein expression (high vs. low) in GC measured by IHC. Thus, through the selection procedure, we finally obtained the 10 included studies which determined RHOA protein expression in GC by IHC, along with clinical features. Each study reported RHOA high and low expressing groups in GC, according to IHC scores. Table 1 summarized the 10 publications.

### Statistical correlations between RHOA expression and clinicopathological features in GC

**Fig. 2:**
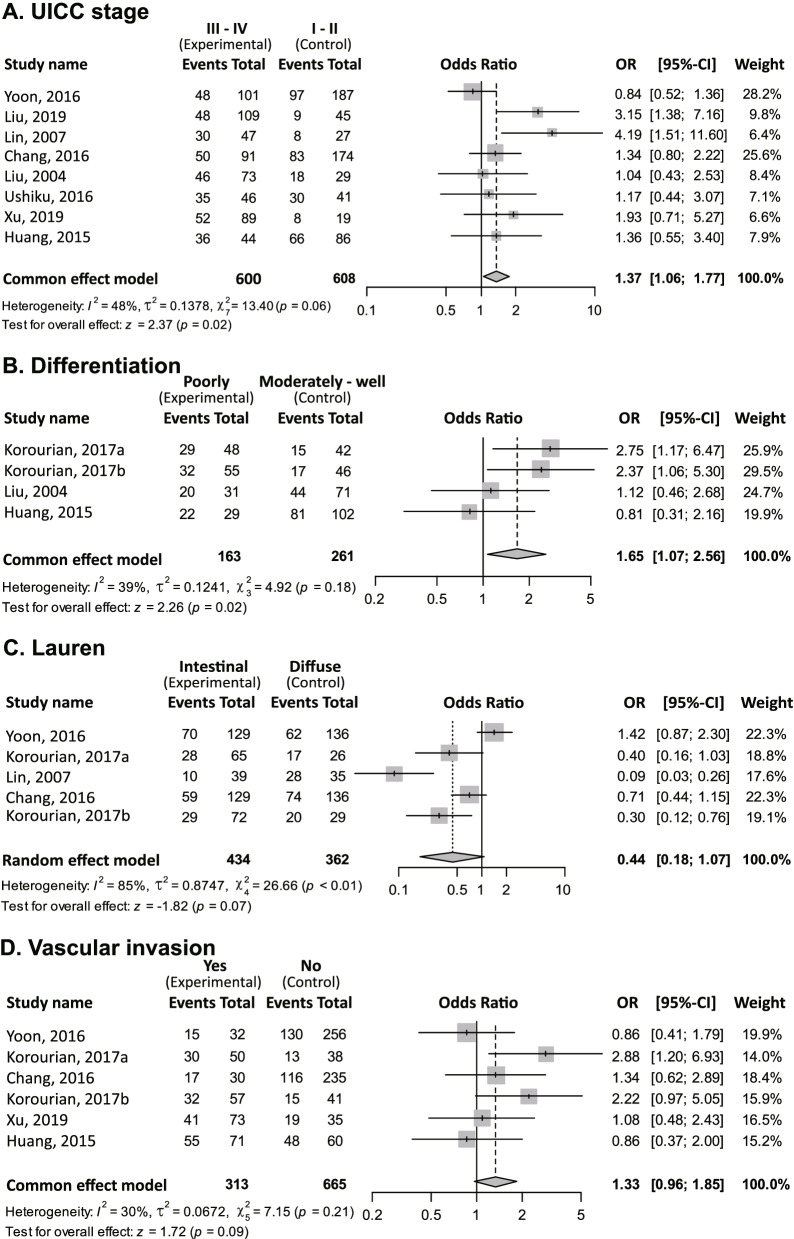
Meta-analysis on clinical parameters. The first column indicates study names; the second column indicates experimental group; the third column indicates control group; the fourth column indicates forest plot; the fifth column indicates odds ratios (ORs) of RHOA protein high- vs. low-expressing patients in the experimental group vs. the control group, and 95% confidence intervals (CI); and the sixth column indicates weight. Events indicate RHOA protein high expressing patients with GC (equivalently, RHOA positive patients with GC). Given a clinical feature, one overall pooled effect size of OR for RHOA high- and low-expressing patients was obtained. Also, heterogeneity was measured by between-study variance τ^2^, Higgins’ I^2^ and Cochran’s Q-tests. **A** Union for International Cancer Control (UICC) stages III–IV (experimental group) vs. I–II (control group). In each group, events (i.e., RHOA protein high-expressing patients with GC) were obtained from each study. The overall effect estimate indicates that the OR of RHOA protein high expression over low expression between the two groups was greater than one. Thus, RHOA protein high expressing patients in the experimental group (i.e., stages III–IV) are more prevalent than in the control group (I–II). In other words, RHOA protein positivity is likely to be advanced UICC stages (i.e., UICC stage progression). **B** Poorly vs. “well plus moderately differentiated” types. **C** Lauren subtypes diffuse vs. Lauren intestinal. **D** Vascular invasion statuses of yes vs. no

RHOA expression positivity was significantly associated with UICC stage progression (OR [III–IV vs. I–II] = 1.37; 95% CI = 1.06–1.77; *P* = 0.02; fixed-effect; Fig. 2A).

Positive RHOA expression was significantly associated with poorly differentiated status (OR [poorly differentiated vs. well or moderately differentiated] = 1.65; 95% CI = 1.07–2.56; *P* = 0.02; fixed-effect; Fig. 2B).

RHOA positivity was not statistically significantly associated with Lauren classification (OR [Lauren intestinal vs. diffuse subtypes] = 0.44; 95% CI = 0.18–1.07; *P* = 0.07; random effect; Fig. 2C). The fixed effect model for the association between RHOA expression and Lauren classification revealed statistical significance (OR [Lauren intestinal subtype vs. Lauren diffuse subtype] = 0.68; 95% CI = 0.51–0.91; *P* = 0.01; fixed-effect). Notably, the fixed effect model results require careful interpretation due to heterogeneity (heterogeneity test, *P* < 0.01). The association between RHOA positivity and vascular invasion was not statistically significant (*P* = 0.09; Fig. 2D). The other clinical features (i.e., sex, age, UICC T classification, UICC M classification, UICC N classification, Bormann types, lymphatic invasion, neural invasion, and tumor sites) were not statistically significantly associated with RHOA protein high- vs. low-expressing patients with GC.

The *P* values of the Q-tests for UICC stage progression, poorly differentiated status, and vascular invasion status were 0.06, 0.18 and 0.21, respectively (Figs. 2A, B, and D). Thus, the null hypotheses that the effect sizes are equal in all studies were not rejected, indicating the effect sizes did not vary across studies. However, for Lauren subtypes (Fig. 2C), the null was rejected (*P* < 0.01), and between-study variance could not be ignored.

### Sensitivity analysis and publication bias

**Fig. 3:**
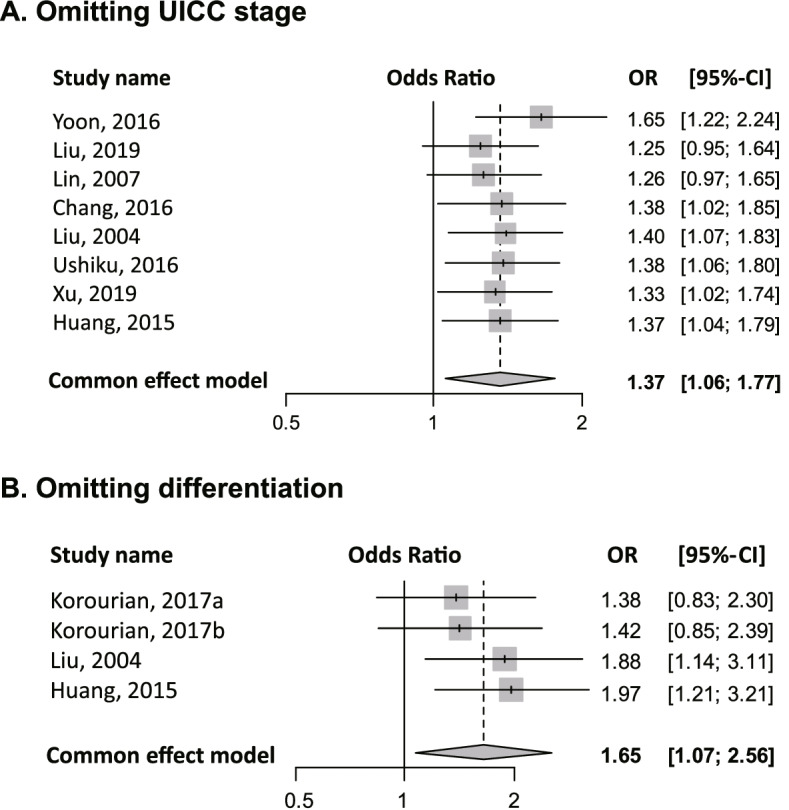
Sensitivity analyses of the meta-analysis results. **A** UICC stages III–IV vs. I–II. **B** Poorly vs. “well plus moderately differentiated” types

For sensitivity analysis of UICC stage progression (Fig. 3A), the ORs were unchanged. Furthermore, sensitivity analysis revealed no significant changes in the ORs for poorly differentiated status (Fig. 3B). Resultingly, sensitivity analysis for UICC stage progression and poorly differentiated status supported the robustness of the conclusion.

**Fig. 4:**
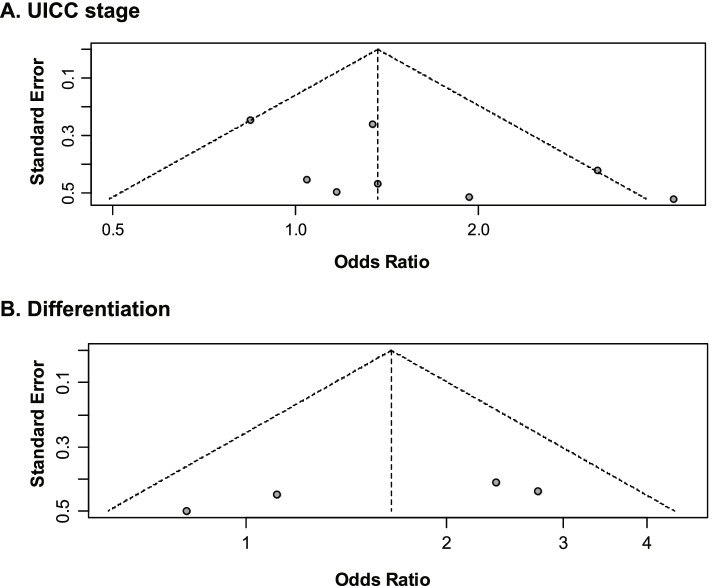
Funnel plots for inspecting publication biases. **A** UICC stages III–IV versus I–II. **B** Poorly vs. “well plus moderately differentiated” types

The visual inspection for the funnel plot of UICC stage progression (Fig. 4A) showed slight asymmetry, implicating possible existence of publication bias. For the funnel plot of poorly differentiated phenotype (Fig. 4B), symmetry was observed, indicating no publication bias.

## Discussion

Our research question was to inspect whether clinical features were linked with RHOA protein high- vs. low-expressing patients with GC. Our key finding in this meta-analysis was that RHOA protein-high expressing patients with GC were statistically significantly associated with UICC stage progression, and poorly differentiated status. Our findings suggest that RHOA protein expression is a GC biomarker candidate. Our findings also need to be validated by large prospective cohort studies to secure reproducibility in future.

Regarding associations between protein expression and clinical features in GC, a few recent meta-analysis studies were available for Sp1, CD133 and heparanase [31–33]. To the best of our knowledge, our study is the first meta-analysis of associations between RHOA protein expression and GC clinical features.

RHOA has emerged as a functionally important molecule in GC [7]. RHOA knockdown revealed anticancer effects in GC cell lines and xenograft models, indicating its potential as a therapeutic target in GC [7]. In in vitro and xenograft models, small molecular weight drugs directly bind to the RHOA protein, repressing RHOA signaling [7]. In fact, reduced cell viability was demonstrated in our novel RHOA inhibitors, JK-122, -136, and -139 [7, 8].

RHOA has been identified as a mediator of EMT [34, 35]. EMT also contributes to tumor progression in the later stages [36]. Thus, in this meta-analysis, the link between UICC stage development and RHOA positivity may be consistent with RHOA-related EMT [7].

EMT is involved in cellular morphology changes, mainly by blocking differentiation-related genes [36]. Therefore, this is consistent with the statistical associations between RHOA positivity and poorly differentiated GC in our meta-analysis. Also, EMT is involved in GC progression of Lauren diffuse subtype [37]. Considering the role of RHOA in EMT [34, 35], the association between Lauren subtypes and RHOA protein expression would been expected, but the association was not significant (Fig. 2C). Another systematic review on the association between RHOA protein expression and Lauren subtypes will be required if the number of RHOA-related studies increases in the future.

RhoA signaling is crucial for two clinical aspects [5, 38, 39]: (1) possible biomarkers and (2) therapeutic target possibilities, based on the aforementioned functional roles of RHOA and our meta-analysis findings. Meta-analyses can provide insights for the usage of RHOA for patient classification.

### Strength of the current study

Our meta-analysis has strengths. We believe that the included studies for the systematic review and meta-analysis cover a comprehensive collection of RHOA protein expression measured by IHC in the field of GC. In addition, 1,389 patients with GC in the ten studies provides statistical strengths for robust meta-analyses.

### Limitations of the meta analysis

There are limitations in this study. Since our meta-analysis utilized published studies, publication bias is unavoidable, indicating statistical heterogeneity is inevitable [40]. The diverse source of the patients in the selected studies and RHOA protein expression measurements by different IHC scoring scheme may affect publication bias. In addition, RHOA antibodies (Table 1) for staining the protein were different through the selected studies, which may also result in publication bias.

## Conclusions

Our study suggests that high RHOA protein expression is associated with.

UICC stage progression (i.e., advanced UICC stages) and poorly differentiated status in patients with GC. However, our study suggests the need of prospective large-scale cohort studies for validation, which helps to prove feasibility of RHOA protein expression as a biomarker to predict GC progression.

## Data Availability

All data are included in this article.

## Abbreviations

GC: Gastric cancer
RHOA: Ras homologous A
EMT: Epithelial-to-mesenchymal transition (EMT)
CNAs: Copy number alterations [CNAs]
GS: Genomically stable” (GS)
IHC: Immunohistochemistry (IHC)
ORs: Odds ratios
Cis: Confidence intervals
UICC: Union for International Cancer Control
